# Dendritic Cell Response to HIV-1 Is Controlled by Differentiation Programs in the Cells and Strain-Specific Properties of the Virus

**DOI:** 10.3389/fimmu.2017.00244

**Published:** 2017-03-13

**Authors:** Aikaterini Nasi, Sylvie Amu, Mårten Göthlin, Marianne Jansson, Noemi Nagy, Francesca Chiodi, Bence Réthi

**Affiliations:** ^1^Department of Microbiology, Tumor and Cell Biology, Karolinska Institutet, Stockholm, Sweden; ^2^Department of Laboratory Medicine, Lund University, Lund, Sweden; ^3^Department of Medicine, Solna (MedS), Karolinska Institutet and Karolinska Universitetssjukhuset, Stockholm, Sweden

**Keywords:** HIV-1, dendritic cell, cytokines, IL-12, infection

## Abstract

Dendritic cells (DCs) are potent antigen-presenting cells that might play contradictory roles during HIV-1 infection, contributing not only to antiviral immunity but also to viral dissemination and immune evasion. Although DCs are characterized by enormous functional diversity, it has not been analyzed how differentially programmed DCs interact with HIV-1. We have previously described the reprogramming of DC development by endogenously produced lactic acid that accumulated in a cell culture density-dependent manner and provided a long-lasting anti-inflammatory signal to the cells. By exploiting this mechanism, we generated immunostimulatory DCs characterized by the production of TH1 polarizing and inflammatory mediators or, alternatively, suppressed DCs that produce IL-10 upon activation, and we tested the interaction of these DC types with different HIV-1 strains. Cytokine patterns were monitored in HIV-1-exposed DC cultures. Our results showed that DCs receiving suppressive developmental program strongly upregulated their capacity to produce the TH1 polarizing cytokine IL-12 and the inflammatory chemokines CCL2 and CCL7 upon interaction with HIV-1 strains IIIB and SF162. On the contrary, HIV-1 abolished cytokine production in the more inflammatory DC types. Preincubation of the cells with the HIV-1 proteins gp120 and Nef could inhibit IL-12 production irrespectively of the tested DC types, whereas MyD88- and TRIF-dependent signals stimulated IL-12 production in the suppressed DC type only. Rewiring of DC cytokines did not require DC infections or ligation of the HIV-1 receptor CD209. A third HIV-1 strain, BaL, could not modulate DC cytokines in a similar manner indicating that individual HIV-1 strains can differ in their capacity to influence DCs. Our results demonstrated that HIV-1 could not induce definite and invariable modulatory programs in DCs. Instead, interaction with the virus triggered different responses in different DC types. Thus, the outcome of DC-HIV-1 interactions might be highly variable, shaped by endogenous features of the cells and diversity of the virus.

## Introduction

Due to their profound functional diversity, dendritic cells (DCs) can potentiate various types of immune reactions ranging from inflammatory TH1 responses against intracellular pathogens to the establishment of antigen-specific tolerance. Such functional plasticity can be exploited by pathogens, including HIV-1, to achieve immune evasion (1). DCs can bind, preserve, and transfer infective virions to CD4^+^ lymphocytes, which might facilitate HIV-1 dissemination both at the mucosal sites of infection and inside peripheral lymphoid tissues (2). In addition, rewiring DC functions might help HIV-1 to dampen antiviral immunity, and, indirectly, it can also decrease responses against non-HIV-related antigens, potentially influencing the outcome of vaccinations or immunotherapies in HIV-1-infected individuals.

Dendritic cells are equipped with an array of delicate pattern recognition receptor (PRR) systems for invading pathogens, as exemplified by the high number of molecules binding HIV-1 or viral compounds including lectins such as CD209 (DC-SIGN), SIGLEC1, mannose receptor or DCIR, the CD4 molecule, the TAM receptors or sensors for viral nucleotides including cGAS in the cytosol, and toll-like receptors (TLRs) in the endosomes. In response to PRR activation, DCs upregulate costimulatory and MHC molecules, which facilitate antigen presentation, and migrate to peripheral lymphoid tissues producing soluble factors that increase inflammation and regulate the differentiation of helper T cells. This scenario might be altered upon encountering HIV-1 as DCs can be either directly infected by the virus, albeit at a relatively low efficiency, or the cells can also be affected in a bystander manner by the binding of various HIV-1-derived compounds (1). Infection of DCs has been shown to increase the costimulatory potential and the ability of the cells to induce T cell activation through an autocrine loop of type-I IFN-mediated DC activation (3, 4). Upregulation of costimulatory molecules in HIV-1-treated DCs has been detected in several studies (5–7); however, it has also been demonstrated that the infection of DCs by HIV-1 inhibited the production of IL-12, the key cytokine supporting TH1 responses (5). Both the HIV-1 envelope protein gp120 and the viral protein R have been implicated in the inhibition of IL-12 production (8, 9), and additionally both compounds contributed to an increased production of the immunosuppressive cytokine IL-10 (9, 10). Controversially to the aforementioned findings, it has also been shown that HIV-1 infection inhibits costimulatory molecule expressions in DCs (11) and HIV-1 binding to CD209 molecules increased IL-12 gene expression in activated DCs (12).

Such often-contradictory findings on HIV-1-mediated stimulatory and inhibitory signals in DCs potentially reflect the concomitant activation of antiviral immune mechanisms and HIV-1-specific immunosuppressive signals in the cells. Nevertheless, diversity in the experimental systems and virus preparations might also contribute to variable results. In this study, we decided to evaluate the impact of DC heterogeneity on responses to HIV-1. We exploited an endogenous, lactic acid-mediated mechanism in developing DC cultures to generate DCs with strong inflammatory and T cell stimulatory potential or, alternatively, suppressed DCs characterized by robust IL-10 production (13, 14) and we tested the interaction of these cells with HIV-1. Our results indicated radically opposing responses in the two DC types upon encountering HIV-1. The virus strains IIIB and SF162, although presented little infectivity in DC cultures, strongly upregulated the secretion of IL-12, CCL2, and CCL7 in suppressed DCs, whereas these virus strains abrogated cytokine production in the more immunostimulatory DC types. HIV-1 BaL, on the contrary, had no impact on cytokine production indicating that strain-specific features might also influence DC-HIV interactions. Our results thus indicated a previously unnoticed high level of complexity in HIV-1 DC interactions, where DC endogenous mechanisms determined largely the response to virus binding. These findings highlighted the need for more in-depth studies on HIV-1 interactions including different *in vivo* existing DC populations and variable virus strains, to understand better the role of DCs in HIV-1 pathogenicity.

## Materials and Methods

### Generation of Monocyte-Derived DCs

The study was performed in accordance to ethical permit approved by the ethical committee at Karolinska Institutet. Blood samples (buffy coats) from healthy donors were collected at the Karolinska Hospital. Ethical permission was needed to use human cells for our study but consent from blood donors about the specific purpose of the experimental work using these buffy coats was not required. Monocytes were isolated from peripheral blood mononuclear cells (PBMCs) using CD14 microbeads (Miltenyi Biotec, Bergisch Gladbach, Germany) after Ficoll gradient centrifugation. Monocytes were cultured at cell culture concentrations of 2 × 10^6^ cells/ml or 0.2 × 10^6^ cells/ml in the presence of 50 ng/ml IL-4 (Peprotech, London, UK) and 75 ng/ml GM-CSF (Gentaur, Kampenhout, Belgium) in RPMI 1640 medium supplemented with antibiotics and 10% FCS (Life Technologies). By using dense and sparse cultures, we utilized a cell culture density-dependent differentiation switch in the developing cells and generated DCs with unique cytokine profiles [(13); Figure S1 in Supplementary Material]. On day 3, the cells were collected and counted using trypan blue exclusion. For DC activation, 250 ng/ml LPS (Invivogen, CA, USA) was used. HIV-1 envelope glycoproteins and Nef were obtained from the NIH AIDS reagent program, and *Mycobacterium tuberculosis* mannosilated lipoarabinomannan (ManLAM) was kindly provided by Andrzej Pawlowski, Lund Universiy, Lund, Sweden. Endotoxin contamination was tested using the THP-1-XBlue-MD2-CD14 bioassay system (Invivogen).

### HIV-1 Propagation and Treatment of DC Cultures

The virus strains SF162, IIIB, and BaL were propagated in PBMC cultures activated by 2.5 μg/ml phytohemagglutinin (PHA) and 10 U/ml IL-2 (both from Sigma-Aldrich, St. Louis, MO, USA). Virus-free control supernatants were also generated using the same PBMC culture conditions. Virus and control preparations were concentrated 50× and thereafter washed in 50× volume PBS, using 100 kDa MW centrifugation filters (Merck Milipore, Billerica, MA, USA). Tissue culture 50% infectious doses (TCID50) were determined using PBMC cultures activated by PHA and IL-2 and treated with serial virus stock dilutions in six replicates. HIV-1 infection was monitored in these cultures on day 7, following addition of the virus, using p24 ELISA (Biomerieux, Marcy Letoile, France). TCID50 was calculated using the Spearman and Karber algorithm (15). For treatment of DC cultures, 100 TCID50 of each virus isolate was used. The cells were treated for 24 h with the viruses or respective control preparations followed by removal of supernatant and activation with LPS for additional 24 h. For detailed fractionation of the HIV-1 and control supernatants, we first used 300-kDa centrifugation filters (Sigma-Aldrich), followed by the subsequent centrifugation of the flow-through fractions using 30-kDa filters. In some experiments, DCs were treated with HIV-1 in the presence of 2.5 μg/ml AZT (Sigma-Aldrich) or inhibitors of MyD88- and TRIF-mediated signals (Invivogen) used in the concentration of 25 μM.

### DC Infection Experiments

Dendritic cells were treated with the virus or control preparations for 24 h, then washed, and cultured for 7 days in the presence of GM-CSF and IL-4. Allogeneic CD4^+^ T cells were enriched from buffy coats using the CD4^+^ T cell isolation kit (Miltenyi Biotec) and added to some of the cultures in 1:3 (DC:T cell) ratio together with 10 U/ml IL-2 (Sigma-Aldrich). To detect DC infection, intracellular stainings were performed with the anti-p24 KC57-RD1 antibody (Beckman Coulter, Brea, CA, USA), using 4% paraformaldehyde fixative and Perm/Wash buffer (BD Biosciences), and the samples were analyzed using flow cytometry.

### Cytokine Measurements

For measurement of cytokine concentration, the following ELISA kits were used: human TNF-α ELISA MAX deluxe set (Biolegend, San Diego, CA, USA), human MCP3/CCL7 ELISA kit (Sigma-Aldrich), human CCL-2 ELISA Ready-SET-Go from Affymetrix e-Bioscience (San Diego, CA, USA), human IL-6 and IL-10 DuoSet ELISAs (R&D, Minneapolis, MN, USA), and human IL-12 (p70) ELISA set from BD Biosciences.

### Flow Cytometry

FITC-labeled anti-CD80, PE-labeled anti-CD86, PE-Cy5-labeled anti-CD83, and APC-conjugated anti-CD209 and anti-CD95 antibodies were obtained from BD Pharmingen (San Diego, CA, USA). Dead cells were stained using the Live/Death detection kit with a near-infrared dye (Invitrogen, Carlsbad, CA, USA). The samples were analyzed using CyAn ADP Analyser (Beckman Coulter, Brea, CA, USA), and the data were analyzed using FlowJo version 9.2 (Tree Star Inc., Ashland, OR, USA).

### CD209 Silencing

CD209-specific and control siRNA were obtained from Applied Biosystems. Electroporations were performed in opti-MEM medium (Invitrogen) in 4-mm cuvettes (Biorad, Hercules, CA, USA) using the GenePulser X cell from Biorad. The cells were then cultured for 48 h in the presence of siRNA, and CD209 expression was analyzed using flow cytometry.

### Statistical Analysis

We used parametric (one-way ANOVA with Tukey’s posttest) and non-parametric (Kruskal–Wallis test, Dunn’s posttest) statistical tests when comparing relative cytokine expressions and paired *t*-test or Wilcoxon signed-rank test to compare absolute concentrations, depending on the distribution of the variables. Statistical analyses were performed using Prism (version 5.0a, GraphPad Software Inc., San Diego, CA, USA).

## Results

### HIV-1 Promotes Unique Functional Responses in Different DC Types

**Figure 1:**
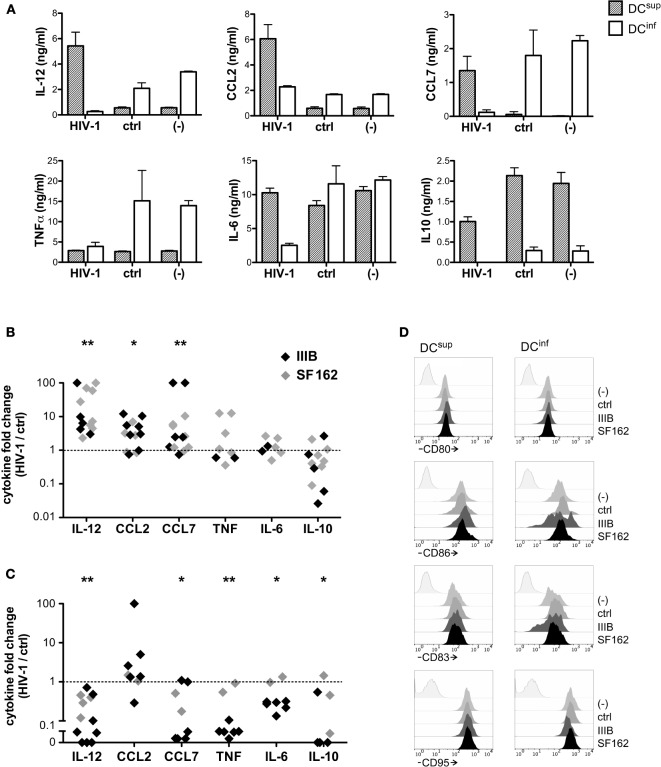
**HIV-1 elicits unique functional responses in different dendritic cell (DC) types**. To analyze how DCs with unique functional profiles interact with HIV-1, we generated inflammatory DCs, with the capacity to produce high levels of IL-12, CCL2, CCL7 and TNF upon activation or, alternatively, suppressed DCs, characterized by IL-10 production, and we exposed these cells to the HIV-1 strain IIIB or SF162 or a virus-free control preparation for 24 h. Thereafter the DCs were activated by 250 ng/ml LPS in fresh medium for 24 h, and cytokine levels were analyzed in the supernatants. Cytokine concentrations (mean ± SD, calculated from triplicate wells) are shown in one representative experiment, using IIIB **(A)**. Alternatively, cytokine levels detected in virus-treated cultures are expressed following normalization with levels observed in control samples. The symbols represent individual experiments performed with DC^sup^ **(B)** or DC^inf^ **(C)**. We analyzed how preincubation of the different DC types with IIIB and SF162 HIV-1 strains influence the expression of CD80, CD86, CD83, and CD95 molecules in LPS-activated DCs, using flow cytometry **(D)**. Representative results of two independent experiments are shown (*p < 0.05, **p < 0.01, and ***p < 0.001).

To analyze how differentially programmed DC types respond to HIV-1, we utilized a previously characterized cell concentration- and lactic acid-dependent mechanism that allowed us to obtain monocyte-derived inflammatory DCs (DC^inf^) producing high levels of the TH1 polarizing cytokine IL-12 together with the inflammatory mediators TNF, CCL2, and CCL7 or, alternatively, suppressed DCs (DC^sup^) that secreted high amounts of IL-10 upon activation (13) (Figure S1 Supplementary Material). DC-HIV interactions have so far been primarily studied using human monocyte-derived DCs, and therefore, the functional variability achieved in this cell type provided us with an experimental platform that is both comparable and relevant in light of previous findings. Preincubation of the different DC types with the HIV-1 strains SF162 and IIIB (an R5 and X4 strain, respectively) induced substantial reprogramming in cytokine production triggered by the TLR4 ligand LPS (Figures 1A–C). DCs developing in dense cultures acquired a suppressed phenotype during their differentiation; however, in response to HIV-1 exposure, these cells strongly increased their potential to produce IL-12 and the inflammatory chemokines CCL2 and CCL7 (Figures 1A,B). We have also observed a tendency of decreased IL-10 production in HIV-1 treated samples, although this effect did not reach statistical significance. On the contrary to DC^sup^, the production of IL-12, CCL7, TNF, and IL-6 was dampened by HIV-1 in inflammatory DCs, which were originally characterized by secretion of high level of these mediators (Figures 1A,C). IL-10 production by DC^inf^ decreased also by HIV-1 pretreatments, suggesting similar virus-mediated effects on both inflammatory and suppressive mediators. As opposed to the rewiring of DC cytokines, the activation markers CD80, CD86, CD83, and CD95 were not or only modestly affected by HIV-1 preincubation on the different DC types (Figure 1D). In fact, CD83 and CD86 were expressed at a slightly lower level on some of the IIIB pretreated DC^inf^ following the LPS-induced activation, whereas SF162 had no effect on any of the tested markers.

**Figure 2:**
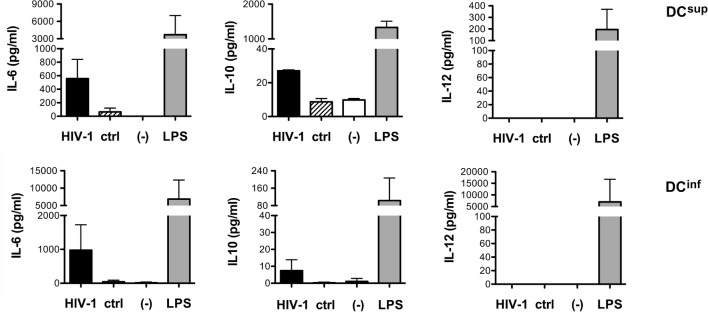
**HIV-1 induced cytokine production in different DC types**. The levels of IL-6, IL-10, and IL-12 were measured following a 24-h treatment of immature DCs with HIV-1 strain SF162, with control preparations or in untreated cell cultures. LPS was used as a positive control in these experiments. Representative results from at least three independent experiments are shown.

The opposing effects of HIV-1 on the cytokine profile of the two functionally different DC lineages might indicate fundamental differences in the HIV-activated receptors and signaling processes. Nevertheless, HIV-1 induced a modest but consistent IL-6 and IL-10 production by both DC^sup^ and DC^inf^, but no secretion of IL-12, suggesting at least partially overlapping immediate responses in the two DC types (Figure 2).

### IL-12 Modulation Is Dependent on Viral Strains

**Figure 3:**
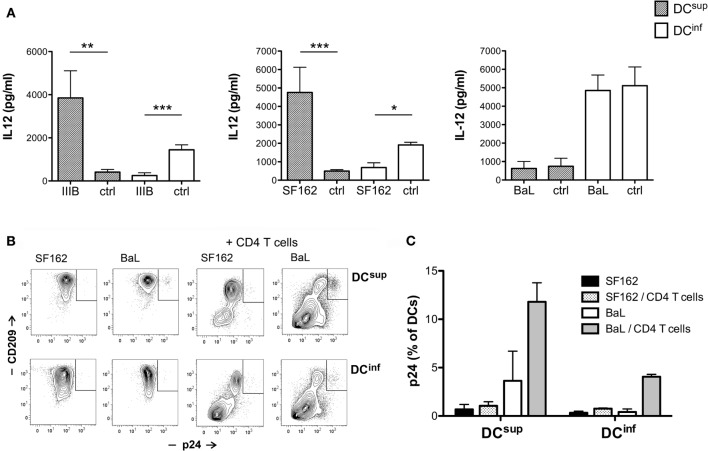
**IL-12 modulation is dependent on viral strains**. **(A)** Immature DCs were exposed to HIV-1 for 24 h, using the strains IIIB, SF162, and BaL or control preparations (ctrl). The cells were then activated by 250 ng/ml LPS in fresh medium for 24 h, and IL-12 levels were analyzed in the supernatants. DC infection was monitored by measuring the intracellular expression of HIV-1 p24 using flow cytometry. DC^sup^ and DC^inf^ were exposed to SF162 or BaL for 24 h, and then the cells were washed and incubated for 6 days in the presence or absence of MACS-purified allogeneic CD4^+^ T lymphocytes. DCs were identified with the help of CD209 staining in the coculture experiments. Representative results are shown with the gated p24^+^ DC population **(B)** in addition to infection levels calculated from three independent experiments **(C)** (*p < 0.05, **p < 0.01, and ***p < 0.001).

Interestingly, although the HIV-1 strains SF162 and IIIB could both induce vigorous changes in DC cytokine production, by boosting IL-12 production in suppressed DCs and inhibiting IL-12 in immunostimulatory DCs, a third HIV-1 strain, BaL, had no effect on IL-12 levels under the same experimental conditions (Figure 3A). HIV-1 BaL has been frequently studied in DC infection experiments, and a higher infectivity has been demonstrated for BaL, compared to IIIB, in DC cultures (16). Therefore, we tested whether the susceptibility of DCs to infection by different HIV-1 strains could be linked to a differential ability of these strains to modulate cytokine production. HIV-1 infection, monitored by measuring the capsid protein p24, was detected in very few (typically <1%) of the SF162-treated DCs, using a virus concentration that efficiently modulated IL-12 production in previous experiments, and in a slightly larger but still minor population of BaL-treated cells (Figures 3B,C). Similar to SF162, the infection of both DC types remained <1% in cultures treated with IIIB (*n* = 3, data not shown). In the presence of allogeneic CD4^+^ T lymphocytes the infection of DCs increased strongly, reaching >10% of the DCs in case of BaL, demonstrating functionality of the studied virus preparations. These results indicated that the modulation of DC cytokines by the SF162 and IIIB strains did not require a productive infection in DCs, and the rewiring of cytokines can occur in a bystander manner at subinfectious viral levels. To further confirm this hypothesis, we treated DC^sup^ and DC^inf^ with HIV-1 IIIB in the presence or absence of 2.5 μg/ml AZT (zidovudine), an inhibitor of reverse transcriptase. Notably, HIV-1 similarly modulated the IL-12 production of DC^sup^ and DC^inf^ in the presence or absence of AZT, suggesting further a bystander effect on the cells (*n* = 3, data not shown).

### Structural Requirements for HIV-1-Mediated Modulation of the Different DC Types

**Figure 4:**
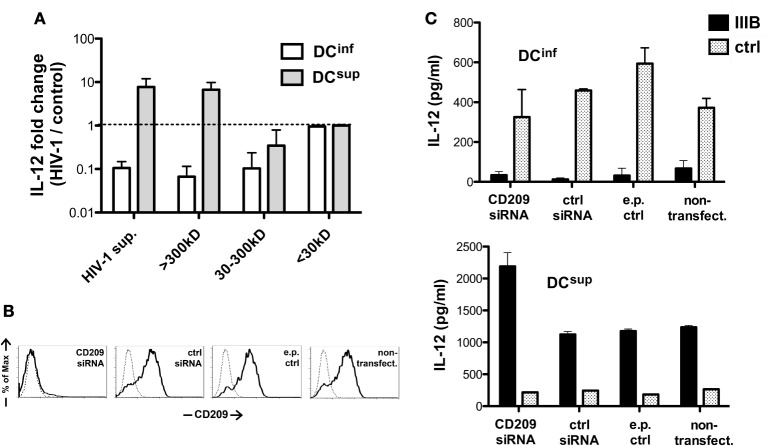
**Structural requirements for HIV-1-mediated DC modulation**. **(A)** DCs were treated with HIV-1 IIIB containing supernatants following MW-based fractionation using membrane filters. The cells were thereafter activated by LPS, and IL-12 levels were measured in the supernatants and normalized with concentration values measured in control samples. Representative results of three independent experiments are shown. **(B)** CD209 molecules were downregulated in DC^sup^ and DC^inf^ by transfecting the cells with CD209-targeting siRNA 2 days prior to HIV-1 treatments. CD209 downregulation was verified by monitoring expression levels following electroporation with CD209, control (ctrl), or no siRNA and in untreated DCs, using flow cytometry (data for DC^inf^ are shown). **(C)** DC^inf^ and DC^sup^ were treated with HIV-1 IIIB for 24 h after siRNA treatments, and the cells were then activated by LPS for 24 h. Representative results show IL-12 concentrations in one of three independent experiments. In these experiments, we used both SF162 and IIIB, with no difference observed between the strains.

To better understand how HIV-1 modulates the different DC types, we utilized a size-based fractionation of the HIV-1 preparations to separate smaller molecular components from virus particles or large macromolecular complexes and we tested the effects of these fractions individually in DC cultures. Treatment of the cells with the molecular weight fraction >300 kDa resulted in similar effects, i.e., stimulation of LPS-induced IL-12 production in DC^sup^ and inhibition of DC^inf^, as the unfractionated HIV-1 supernatants, whereas molecular components in the range between 30 and 300 kD induced an inhibitory signal on DC^inf^, but possessed no stimulatory effects (Figure 4A). Molecules of even smaller size (<30 kDa) had no effect on IL-12 production. These results suggested that the DC modulatory signals are carried by larger components, which can include viral particles, microvesicles associated with viral replication or larger molecule complexes. In addition, soluble viral proteins also contributed to DC inhibitory signals.

In the next set of experiments, we analyzed the role of CD209, one of the major HIV-1 receptors in DCs, which has demonstrated roles in both DC suppression and increased IL-12 transcription upon HIV-1 binding (12, 17). We downmodulated CD209 on the surface of immature DCs using siRNA technology (Figure 4B) before exposing DC^sup^ and DC^inf^ to HIV-1. Interestingly, the lack of CD209 had no effect on the viral inhibition of DC^inf^ or on stimulation of IL-12 production in DC^sup^ (Figure 4C). In fact, levels of IL-12 were slightly elevated in the absence of CD209 in HIV-1 pretreated DC^sup^, suggesting a modest suppressive role for CD209 in IL-12 regulation. These results indicated that the virus-induced CD209 signaling might not be responsible for the differential regulation of cytokine production observed in the two DC types.

### Viral Proteins Downmodulate IL-12 in Both DC Types, and TRIF- and MyD88-Mediated Signals Increase IL-12 in Suppressed DCs Only

**Figure 5:**
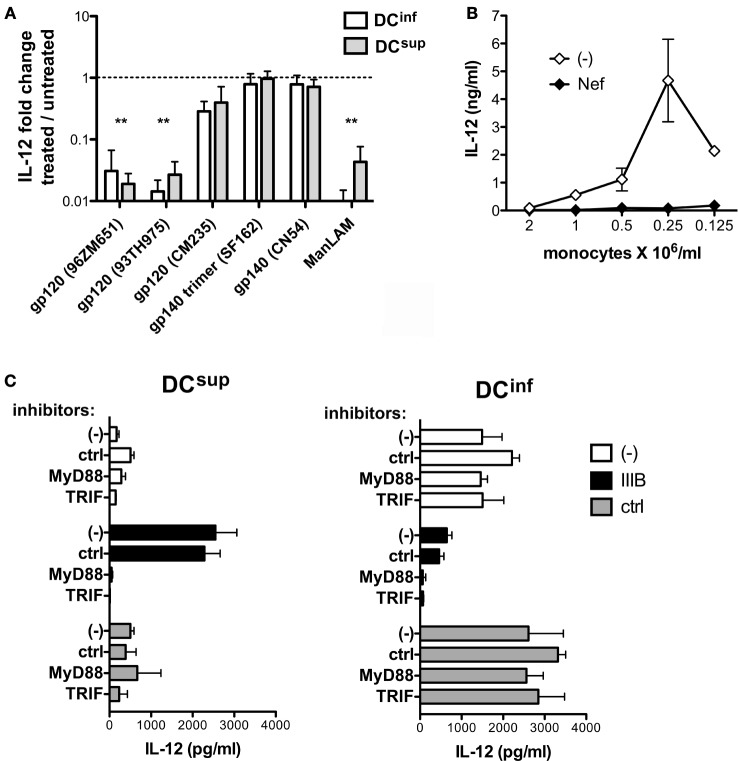
**HIV-1 induced pathways in suppressed and inflammatory DC types**. **(A)** DCs were pretreated with 5 μg/ml gp120 or mycobacterial mannosilated lipoarabinomannan (ManLAM) for 24 h, and the cells were thereafter activated by LPS for 24 h. IL-12 levels were measured in the culture supernatants and were related to samples receiving LPS in the absence of pretreatments. Results are calculated from three independent experiments, **p < 0.01. **(B)** DCs, obtained from cultures representing a range of different cell culture densities were pretreated with 5 μg/ml recombinant Nef for 24 h, and the cells were thereafter activated by LPS. IL-12 concentrations are shown from one of three independent experiments. **(C)** The different DC types were pretreated with MyD88 and TRIF inhibitors for 6 h before the addition of HIV-1 IIIB to the cultures for 24 h. The cells were activated with LPS in fresh medium, and IL-12 production was analyzed in the culture supernatant. Representative results are shown from three independent experiments.

The viral gp120 proteins play essential roles in binding a wide range of target cell receptors, and these molecules can efficiently modulate DC functions (18). We analyzed whether DC binding by gp120 could be sufficient to modulate IL-12 production in DC^sup^ and DC^inf^ by exposing these cells to recombinant gp120 and gp140 molecules representing different X4 and R5 HIV-1 strains before triggering IL-12 production by LPS (Figure 5A). In the same assay, we included purified ManLAM component of *Mycobacterium tuberculosis* that is characterized by similar binding specificity to CD209 as the HIV-1 gp120 (17, 19). The results indicated clear inhibitory signals elicited by gp120 proteins of the strains 96ZM651 and 93TH975 and by ManLAM on IL-12 production in both DC types, whereas the other tested gp120 and gp140 constructs showed no effect on IL-12 production (Figure 5A). Differences in amino acid sequence, conformation, or glycosylation could potentially contribute to a more or less efficient DC modulation by the various recombinant proteins. Endotoxin contamination of the recombinant proteins, on the other hand, was ruled out with the help of a THP-1-XBlue bioassay system (detection limit 0.05 EU/ml). The HIV-1 protein Nef has been described to modulate DC functions acting in a bystander manner or, alternatively, within the infected cells (20). Similar to gp120, preincubation of DCs obtained from dense or sparse cultures with recombinant Nef resulted in a profound inhibition of the ability of the cells to produce IL-12 in response to a later LPS activation (Figure 5B). HIV-infected cells release Nef in microvesicles that co-exist with viral particles in the HIV-1 preparations and that can be internalized by other cells (21–23). In addition, myeloid cells could be particularly efficient in release Nef, even in the absence of profound viral replication (23), suggesting a potential role for endogenous Nef released by the low number of infected DCs in our culture system. Thus, the envelop protein gp120 and Nef might both contribute to IL-12 inhibition in DCs; however, these molecules may not be sufficient for delivering the signals that upregulate IL-12 production in HIV-1-treated DC^sup^. The endosomal TLRs, TLR3, TLR7, and TLR8 can act as sensors of viral RNA, and these receptors have all been implicated in HIV-1 recognition (24–29). We decided to study the potential contribution of TLR-mediated pathways in the modulation of DC^sup^ and DC^inf^ by HIV-1, with the help of peptide inhibitors that transiently interfere with the dimerization of the MyD88 adapter proteins involved in TLR7 and TLR8 signaling or with the interaction of TLR3 with the TRIF adapter protein. Interestingly, inhibitors of MyD88- and TRIF-signaling could selectively block the stimulatory effects of HIV-1 on IL-12 production of DC^sup^ without interfering with the HIV-1-mediated suppression of DC^inf^ (Figure 5C). These results suggest a concerted action of TLR3 with the MyD88-associated receptors TLR7 and/or TLR8 in the stimulation of DC^sup^ by HIV-1. On the other hand, the same pathway could not stimulate DC^inf^, which might indicate differences in HIV-1 uptake and endosomal transportation in the two DC types.

## Discussion

Modulation of cytokines and costimulatory molecules by HIV-1 has been extensively documented in DCs suggesting altered functions of these cells during HIV-1 infection, which might contribute to viral immune evasion and, somewhat controversially, to an increased immune activation (1, 3–6, 8–12). The description of both inhibitory and stimulatory signals induced by HIV-1, together with the several alternative interpretations for these events, make it complicated to envisage a generalized model for the contribution of DCs in HIV-1 infection and in the following disease progression. In addition, DCs receive unique combination of exogenous differentiation signals and tissue-specific regulatory factors, which can influence their interaction with pathogens. We have previously shown that DC differentiation can be skewed *in vitro* by endogenously produced lactic acid, which accumulated in dense cultures and provided a strong and long-lasting anti-inflammatory stimulus to the cells (13). DCs developing in sparse cultures, on the contrary, avoided the lactate-mediated suppression and produced high levels of inflammatory cytokines, migrated toward lymphoid tissue-derived chemokines, and stimulated the differentiation of TH1 cells (13, 14). This system allowed us to analyze in this study how DCs with unique functional programming respond to HIV-1 encounters. We showed that HIV-1 binding induced a robust reprogramming of the cytokine-producing abilities in DCs, and, surprisingly, the stimulatory or inhibitory nature of the HIV-1 effects was determined by endogenous characteristics of the tested DC types and not by viral compounds. DCs receiving a suppressive developmental program strongly upregulated their potential to produce IL-12, CCL2, and CCL7 in response to HIV-1 exposure, whereas DCs that were already characterized by the production of high levels of these mediators downregulated their cytokine production in response to HIV. Further studies are required to clarify the mechanisms behind the different types of DC responses in the presence of HIV-1; however, our experiments have already revealed several features of the strong DC modulatory effect of HIV-1. The ability of HIV-1 to rewire cytokine production in conditions, which do not allow productive DC infections suggest an effective bystander regulation of DCs and consequently impaired immune responses, even at very low level of virus replication. In our studies, CD209 played a negligible role in cytokine regulation, in spite of the previously demonstrated modulation of IL-12 and IL-10 through CD209-binding ligands (12, 30), suggesting the presence of other important viral pathways acting on DC cytokines. In addition, stimulatory effects of HIV-1 strains IIIB and SF162 on the IL-12 production were not recapitulated by using recombinant gp120 molecules representing the envelope proteins of several HIV-1 strains, including SF162, indicating that the envelope–DC interaction, in itself, might only contribute to inhibitory signals in DCs. Similarly, preincubation of the cells with recombinant Nef resulted in a reduced IL-12 production, irrespective of the DC phenotype. IL-12 upregulation in DC^sup^ appeared to be the consequence of the HIV-1-mediated activation of MyD88- and TRIF-mediated signals, which strongly suggests a role for endosomal TLRs; however, it remained to be understood why the same pathway could not contribute to higher cytokine levels in DC^inf^.

It is tempting to hypothesize how the observed variability of DC-HIV interactions might influence immunity in HIV-1 infected individuals. DCs from dense cultures lacked immunostimulatory properties and in this respect resembled steady-state tissue resident DC types that promote tolerance instead of immune activation. Coincubation with HIV-1 appeared to provide weak TLR stimulation in these DCs boosting their ability to produce various inflammatory cytokines in response to a second activation signal received through TLR4. Such two-stage activation process might be essential for reprogramming cytokine expressions as the same cells secreted minute amounts of inflammatory cytokines, but very high level of IL-10, when TLR4 was activated without previous HIV-1 encounters. Thus, our results suggest that DCs developing in a tolerogenic environment might be efficient in inducing antiviral responses; however, the cells require sequential, gradually increasing activation signals, e.g., through viral compounds, inflammatory cytokines, or T cell interactions, to successfully shift toward an immunostimulatory phenotype. This hypothesis is in line with previous models suggesting powerful synergism between certain TLR ligands (31) or a need for sequential stimulation *via* different activation pathways to avoid functional exhaustion (32). Indeed we could demonstrate that, on the contrary to the suppressed DC type, the more inflammatory DCs rapidly developed functional exhaustion in the presence of persisting viral signals.

On the contrary to acute immune responses, DC activation might be impaired with the HIV-1 infection becoming chronic. Our data suggest that the chronic exposure to HIV-1 particles might lead to persistently perturbed activation threshold in tissue-resident DCs, potentially promoting increased responses to weak and irrelevant activation signals. Successful antiretroviral therapy may correct the HIV-1-mediated rewiring of DCs; however, other bystander events associated with chronic HIV-1 infection, e.g., microbial translocation or the dysregulation of cytokine levels, might be prevailing the suppression of virus replication and could also contribute to hypersensitivity of tissue-resident DCs.

In addition to the variable effects of HIV-1 observed in the different DC types, we have also observed that the HIV-1 strain BaL, unlike IIIB or SF162, could not influence cytokine responses in DCs suggesting a heterogeneity between virus strains in their capacity to reprogram DC activities. It remains to be clarified, potentially by testing larger repertoire of HIV-1 strains or virus combinations isolated from different individuals, whether a variability in DC modulation by the prevailing virus strains could exist between different patients or whether the capacity to control DC cytokines would represent a stable selection criteria universally maintained during the emergence of new virus variants.

In summary, we have described a robust regulation of DC cytokines at subinfectious HIV-1 levels, and our results highlighted the importance of considering DC heterogeneity for better understanding the interaction of DCs and HIV-1. Variability has also been detected between different HIV-1 strains in their ability to modulate DC cytokines, which suggests potentially existing differences in DC-HIV-1 interactions not only between DC types but also between individual patients and different stages of disease progression.

## Author Contributions

AN performed experiments, analyzed the data, and wrote the manuscript. SA and NN performed experiments and critically reviewed the manuscript. MJ and FC provided important research tools and resources and critically reviewed the manuscript. MG performed experiments and analyzed the data. BR designed the project, performed experiments, analyzed the data, and wrote the manuscript.

## Conflict of Interest Statement

The authors declare that the research was conducted in the absence of any commercial or financial relationships that could be construed as a potential conflict of interest.

## References

[1] Miller E Bhardwaj N. Dendritic cell dysregulation during HIV-1 infection. Immunol Rev (2013) 254:170–89. 10.1111/imr.12082 23772620 PMC5590719

[2] Cameron PU Freudenthal PS Barker JM Gezelter S Inaba K Steinman RM. Dendritic cells exposed to human immunodeficiency virus type-1 transmit a vigorous cytopathic infection to CD4+ T cells. Science (1992) 257:383–7. 10.1126/science.1352913 1352913

[3] Manel N Hogstad B Wang Y Levy DE Unutmaz D Littman DR. A cryptic sensor for HIV-1 activates antiviral innate immunity in dendritic cells. Nature (2010) 467:214–7. 10.1038/nature09337 20829794 PMC3051279

[4] Martin-Gayo E Buzon MJ Ouyang Z Hickman T Cronin J Pimenova D Potent cell-intrinsic immune responses in dendritic cells facilitate HIV-1-specific T cell immunity in HIV-1 elite controllers. PLoS Pathog (2015) 11:e1004930. 10.1371/journal.ppat.1004930 26067651 PMC4466270

[5] Smed-Sorensen A Lore K Walther-Jallow L Andersson J Spetz AL. HIV-1-infected dendritic cells up-regulate cell surface markers but fail to produce IL-12 p70 in response to CD40 ligand stimulation. Blood (2004) 104:2810–7. 10.1182/blood-2003-07-2314 15231570

[6] Wilflingseder D Mullauer B Schramek H Banki Z Pruenster M Dierich MP HIV-1-induced migration of monocyte-derived dendritic cells is associated with differential activation of MAPK pathways. J Immunol (2004) 173:7497–505. 10.4049/jimmunol.173.12.7497 15585876

[7] Harman AN Wilkinson J Bye CR Bosnjak L Stern JL Nicholle M HIV induces maturation of monocyte-derived dendritic cells and Langerhans cells. J Immunol (2006) 177:7103–13. 10.4049/jimmunol.177.10.7103 17082627

[8] Fantuzzi L Purificato C Donato K Belardelli F Gessani S. Human immunodeficiency virus type 1 gp120 induces abnormal maturation and functional alterations of dendritic cells: a novel mechanism for AIDS pathogenesis. J Virol (2004) 78:9763–72. 10.1128/JVI.78.18.9763-9772.2004 15331709 PMC515003

[9] Majumder B Janket ML Schafer EA Schaubert K Huang XL Kan-Mitchell J Human immunodeficiency virus type 1 Vpr impairs dendritic cell maturation and T-cell activation: implications for viral immune escape. J Virol (2005) 79:7990–8003. 10.1128/JVI.79.13.7990-8003.2005 15956545 PMC1143734

[10] Shan M Klasse PJ Banerjee K Dey AK Iyer SP Dionisio R HIV-1 gp120 mannoses induce immunosuppressive responses from dendritic cells. PLoS Pathog (2007) 3:e169. 10.1371/journal.ppat.0030169 17983270 PMC2048530

[11] Hertoghs N Van Der Aar AM Setiawan LC Kootstra NA Gringhuis SI Geijtenbeek TB. SAMHD1 degradation enhances active suppression of dendritic cell maturation by HIV-1. J Immunol (2015) 194:4431–7. 10.4049/jimmunol.1403016 25825449

[12] Gringhuis SI Den Dunnen J Litjens M Van Der Vlist M Geijtenbeek TB. Carbohydrate-specific signaling through the DC-SIGN signalosome tailors immunity to *Mycobacterium tuberculosis*, HIV-1 and *Helicobacter pylori*. Nat Immunol (2009) 10:1081–8. 10.1038/ni.1778 19718030

[13] Nasi A Fekete T Krishnamurthy A Snowden S Rajnavolgyi E Catrina AI Dendritic cell reprogramming by endogenously produced lactic acid. J Immunol (2013) 191:3090–9. 10.4049/jimmunol.1300772 23956421

[14] Nasi A Rethi B. Disarmed by density: a glycolytic break for immunostimulatory dendritic cells? Oncoimmunology (2013) 2:e26744. 10.4161/onci.26744 24575378 PMC3926870

[15] Japour AJ Mayers DL Johnson VA Kuritzkes DR Beckett LA Arduino JM Standardized peripheral blood mononuclear cell culture assay for determination of drug susceptibilities of clinical human immunodeficiency virus type 1 isolates. The RV-43 Study Group, the AIDS Clinical Trials Group Virology Committee Resistance Working Group. Antimicrob Agents Chemother (1993) 37:1095–101. 8517697 10.1128/aac.37.5.1095 PMC187907

[16] Smed-Sorensen A Lore K Vasudevan J Louder MK Andersson J Mascola JR Differential susceptibility to human immunodeficiency virus type 1 infection of myeloid and plasmacytoid dendritic cells. J Virol (2005) 79:8861–9. 10.1128/JVI.79.14.8861-8869.2005 15994779 PMC1168781

[17] Geijtenbeek TB Den Dunnen J Gringhuis SI. Pathogen recognition by DC-SIGN shapes adaptive immunity. Future Microbiol (2009) 4:879–90. 10.2217/fmb.09.51 19722841

[18] Chougnet C Gessani S. Role of gp120 in dendritic cell dysfunction in HIV infection. J Leukoc Biol (2006) 80:994–1000. 10.1189/jlb.0306135 16912071

[19] Geijtenbeek TB Van Vliet SJ Koppel EA Sanchez-Hernandez M Vandenbroucke-Grauls CM Appelmelk B Mycobacteria target DC-SIGN to suppress dendritic cell function. J Exp Med (2003) 197:7–17. 10.1084/jem.20021229 12515809 PMC2193797

[20] Quaranta MG Mattioli B Giordani L Viora M. Immunoregulatory effects of HIV-1 Nef protein. Biofactors (2009) 35:169–74. 10.1002/biof.28 19449444

[21] Campbell TD Khan M Huang MB Bond VC Powell MD. HIV-1 Nef protein is secreted into vesicles that can fuse with target cells and virions. Ethn Dis (2008) 18:S2–14. 18646314 PMC3418053

[22] Muratori C Cavallin LE Kratzel K Tinari A De Milito A Fais S Massive secretion by T cells is caused by HIV Nef in infected cells and by Nef transfer to bystander cells. Cell Host Microbe (2009) 6:218–30. 10.1016/j.chom.2009.06.009 19748464

[23] Raymond AD Campbell-Sims TC Khan M Lang M Huang MB Bond VC HIV Type 1 Nef is released from infected cells in CD45(+) microvesicles and is present in the plasma of HIV-infected individuals. AIDS Res Hum Retroviruses (2011) 27:167–78. 10.1089/aid.2009.0170 20964480 PMC3064529

[24] Breckpot K Escors D Arce F Lopes L Karwacz K Van Lint S HIV-1 lentiviral vector immunogenicity is mediated by toll-like receptor 3 (TLR3) and TLR7. J Virol (2010) 84:5627–36. 10.1128/JVI.00014-10 20237085 PMC2876620

[25] Gringhuis SI Van Der Vlist M Van Den Berg LM Den Dunnen J Litjens M Geijtenbeek TB. HIV-1 exploits innate signaling by TLR8 and DC-SIGN for productive infection of dendritic cells. Nat Immunol (2010) 11:419–26. 10.1038/ni.1858 20364151

[26] Sironi M Biasin M Cagliani R Forni D De Luca M Saulle I A common polymorphism in TLR3 confers natural resistance to HIV-1 infection. J Immunol (2012) 188:818–23. 10.4049/jimmunol.1102179 22174453

[27] Huik K Avi R Pauskar M Kallas E Jogeda EL Karki T Association between TLR3 rs3775291 and resistance to HIV among highly exposed Caucasian intravenous drug users. Infect Genet Evol (2013) 20:78–82. 10.1016/j.meegid.2013.08.008 23962581 PMC4001117

[28] Chattergoon MA Latanich R Quinn J Winter ME Buckheit RW III Blankson JN HIV and HCV activate the inflammasome in monocytes and macrophages via endosomal toll-like receptors without induction of type 1 interferon. PLoS Pathog (2014) 10:e1004082. 10.1371/journal.ppat.1004082 24788318 PMC4006909

[29] Guo H Gao J Taxman DJ Ting JP Su L HIV-1 infection induces interleukin-1beta production via TLR8 protein-dependent and NLRP3 inflammasome mechanisms in human monocytes. J Biol Chem (2014) 289:21716–26. 10.1074/jbc.M114.566620 24939850 PMC4118130

[30] Gringhuis SI Den Dunnen J Litjens M Van Het Hof B Van Kooyk Y Geijtenbeek TB. C-type lectin DC-SIGN modulates toll-like receptor signaling via Raf-1 kinase-dependent acetylation of transcription factor NF-kappaB. Immunity (2007) 26:605–16. 10.1016/j.immuni.2007.03.012 17462920

[31] Napolitani G Rinaldi A Bertoni F Sallusto F Lanzavecchia A. Selected toll-like receptor agonist combinations synergistically trigger a T helper type 1-polarizing program in dendritic cells. Nat Immunol (2005) 6:769–76. 10.1038/ni1223 15995707 PMC3760217

[32] Abdi K Singh NJ Matzinger P Lipopolysaccharide-activated dendritic cells: “exhausted” or alert and waiting? J Immunol (2012) 188:5981–9. 10.4049/jimmunol.1102868 22561154 PMC3370068

