# Supplementary material for: Dendritic Cell Response to HIV-1 Is Controlled by Differentiation Programs in the Cells and Strain-Specific Properties of the Virus
Source: Front Immunol. 2017 Mar 13;8:244. doi: 10.3389/fimmu.2017.00244 (PMC5346539; doi:10.3389/fimmu.2017.00244)
Supplement: Supplementary file 1 [file Image_1.PDF]

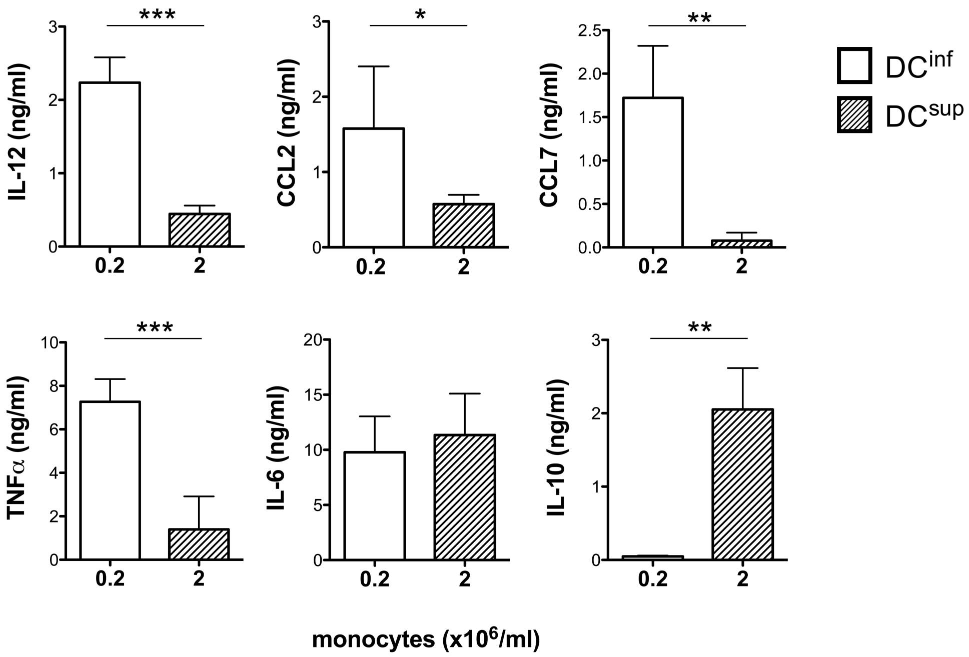

Supplementary figure 1. Dense or sparse cultures potentiate distinct cytokine profiles in monocyte-derived DCs. We introduced a cell culture density-dependent differentiation switch in developing DCs, and generated inflammatory DCs using sparse cultures ($0.2X10^6$ monocytes/ml), which produced high levels of IL-12, CCL2, CCL7 and TNF cytokines in response to 24-hour LPS stimulation or, alternatively, suppressed DCs in dense cultures ($2X10^6$ monocytes/ml), which produced high levels of IL-10 upon activation. IL-6 was secreted similarly by the two DC types. Representative results are shown from >7 independent experiments.
